# Widespread Disruption of Functional Brain Organization in Early-Onset Alzheimer’s Disease

**DOI:** 10.1371/journal.pone.0102995

**Published:** 2014-07-31

**Authors:** Sofie M. Adriaanse, Maja A. A. Binnewijzend, Rik Ossenkoppele, Betty M. Tijms, Wiesje M. van der Flier, Teddy Koene, Lieke L. Smits, Alle Meije Wink, Philip Scheltens, Bart N. M. van Berckel, Frederik Barkhof

**Affiliations:** 1 Department of Radiology and Nuclear Medicine, VU University Medical Center, Amsterdam, The Netherlands; 2 Department of Neurology/Alzheimercenter Amsterdam, VU University Medical Center, Amsterdam, The Netherlands; 3 Department of Epidemiology and biostatistics, VU University Medical Center, Amsterdam, The Netherlands; 4 Department of Medical Psychology, VU University Medical Center, Amsterdam, The Netherlands; 5 Neuroscience Campus Amsterdam, Amsterdam, The Netherlands; University of Electronic Science and Technology of China, China

## Abstract

Early-onset Alzheimer’s disease (AD) patients present a different clinical profile than late-onset AD patients. This can be partially explained by cortical atrophy, although brain organization might provide more insight. The aim of this study was to examine functional connectivity in early-onset and late-onset AD patients. Resting-state fMRI scans of 20 early-onset (<65 years old), 28 late-onset (≥65 years old) AD patients and 15 “young” (<65 years old) and 31 “old” (≥65 years old) age-matched controls were available. Resting-state network-masks were used to create subject-specific maps. Group differences were examined using a non-parametric permutation test, accounting for gray-matter. Performance on five cognitive domains were used in a correlation analysis with functional connectivity in AD patients. Functional connectivity was not different in any of the RSNs when comparing the two control groups (young vs. old controls), which implies that there is no general effect of aging on functional connectivity. Functional connectivity in early-onset AD was lower in all networks compared to age-matched controls, where late-onset AD showed lower functional connectivity in the default-mode network. Functional connectivity was lower in early-onset compared to late-onset AD in auditory-, sensory-motor, dorsal-visual systems and the default mode network. Across patients, an association of functional connectivity of the default mode network was found with visuoconstruction. Functional connectivity of the right dorsal visual system was associated with attention across patients. In late-onset AD patients alone, higher functional connectivity of the sensory-motor system was associated with poorer memory performance. Functional brain organization was more widely disrupted in early-onset AD when compared to late-onset AD. This could possibly explain different clinical profiles, although more research into the relationship of functional connectivity and cognitive performance is needed.

## Introduction

Alzheimer’s disease (AD) is the most common form of dementia. Age is an important risk factor for developing AD [1]; most patients are diagnosed with AD after the age of 65 years. In such late-onset AD patients, memory problems are most prominent and predate decline in other cognitive domains [2]. The prevalence of AD before the age of 65 years is much lower. In these early-onset AD patients, memory problems appear less frequent, but loss of visuo-constructive functioning, language, attention, executive functions, and apraxia are more prevalent [2], [3], [4]. Early-onset AD patients decline more rapidly, compared to late-onset AD patients [5].

Only few studies have compared *in vivo* measures of pathology and brain function in early-onset versus late-onset AD. Hypo-metabolism, measured with Positron Emission Tomography (PET) and fluorine-18 labeled fluorodeoxyglucose ([^18^F]FDG), was reported to be most pronounced in early-onset AD within parietal brain regions [6], [7], but also in (left) frontal cortex, temporal regions [8] and subcortical areas [9]. Using carbon-11 labeled Pittsburgh Compound-B ([^11^C]PIB) PET [10], increased amyloid-β plaque burden in the parietal cortex was observed in early-onset AD patients [6], although others reported no differences in amyloid load [7]. Posterior brain regions showed more severe slowing of spontaneous oscillatory activity on EEG in early-onset AD patients [11]. Cortical atrophy is also most pronounced in parietal and occipital cortex in early-onset AD patients, whereas the medial temporal lobe is most vulnerable in late-onset AD [12], [13]. These findings indicate an increased vulnerability of parietal and occipital cortex in early-onset AD patients. Together, however, they fail to explain the much more widespread cognitive disturbances observed in early-onset AD.

Resting-state functional magnetic resonance imaging (rs-fMRI) can be used to study functional properties of the brain by detecting spontaneous neuronal activity as localized changes in the blood oxyhemoglobin/deoxyhemoglobin ratio [14]. Spatially distinct brain regions with co-varying rs-fMRI signals are considered to be functionally connected [15]. At rest, brain areas that are functionally connected are referred to as resting-state networks (RSNs). Several RSNs have been consistently found and linked to higher-order cognitive functions, such as executive control and visuo-constructive functioning. Basic cognitive functions such as sensory-motor and auditory processing are also represented as RSNs [16]. As such, altered functional connectivity of RSNs might be related to distinct cognitive problems in early-onset AD. Recently, Gour and colleagues were the first to use rs-fMRI to examine functional connectivity in early-onset and late-onset AD patients [17]. Early-onset AD patients showed lower functional connectivity in bilateral dorso-lateral prefrontal network, which is believed to be involved in executive functions. In contrast, lower functional connectivity in late-onset AD patients when compared to early-onset AD patients was identified in the left anterior-medial temporal network; a network involved in memory.

The aim of this study was to examine functional connectivity in early-onset and late-onset AD patients. To investigate the general effect of aging on functional connectivity, two age-matched control groups were compared. Since very limited data is available describing functional connectivity in early-onset and late-onset AD patients, this was an exploratory study within eight standard RSNs [16]. Consistent with atrophy and amyloid plaque patterns, functional connectivity of the posterior cingulate/precuneus and occipital cortex are likely to be affected in early-onset AD. Functional connectivity in frontal regions could also be affected as was seen with [^18^F]FDG-PET. Functional connectivity in late-onset AD patients was hypothesized to be lower in areas linked to memory function.

## Methods

### Ethics statement

The Ethical Review Board of the VU University Medical Center Amsterdam (VUMC) approved the study. All participants provided written informed consent.

### Participants

MRI data of 48 AD patients and 46 healthy controls was available. This was a subset of a larger sample reported earlier [18], where functional connectivity in AD patients, patients with mild cognitive impairment (MCI) and controls was examined. Patients were recruited from the Amsterdam Dementia Cohort at the Alzheimer Center of the VUMC. All patients underwent a standard diagnostic examination, which included physical and neurological examination, medical history taking, extensive neuropsychological testing, screening laboratory tests, MRI and EEG. Using this information, a multidisciplinary team established the clinical diagnosis. All AD patients met NINCDS-ADRDA criteria [19] for ‘probable AD’. Of the majority of AD patients (n = 41) either [^11^C]PIB PET or Cerebral spinal fluid (CSF) measures were available to confirm AD-pathology. Global cognitive functioning was assessed using the Mini Mental State Examination (MMSE) [20]. Age at time of diagnosis was used to distinguish between early-onset (<65 years) and late onset (≥65 years) AD patients. Controls consisted of family members of patients and volunteers recruited through advertisements posted in the Alzheimer Center and activity centers for the elderly. Exclusion criteria included significant medical, neurological (other than AD), or psychiatric illness; a history of brain damage; and use of non AD-related medication known to influence cerebral function. Same exclusion criteria applied to controls with the addition of subjective memory complaints.

### MRI data acquisition

Images were acquired on a 1.5 Tesla scanner (Siemens Sonata, Erlangen, Germany). A high-resolution T1-weighted magnetization prepared rapid acquisition gradient echo (MPRAGE) image (TR = 2700 ms; TE = 3.97 ms; TI = 950 ms; flip angle = 8°; voxel size 1×1.5×1 mm) was acquired for every patient. In addition, resting state functional scans consisted of 200 T2*-weighted echo planar imaging (EPI) volumes (TR = 2850 ms; TE = 60 ms; flip angle = 90°; voxel size 3.3 mm isotropic). Subjects were instructed to lie still with their eyes closed and not to fall asleep during the resting state scan (although this was not monitored).

### Structural MRI analysis

All MRI analyses were done using FMRIB’s Software (FSL 4.1.9; www.fmrib.ox.ac.uk/fsl [21]. Gray matter (GM) volume, normalized for head size (NGMV), was estimated using SIENAX [22]. In short, non-brain tissue of individual T1-images was removed and the scaling factor between T1-image and standard space (MNI 152) was estimated. Next, tissue segmentation was performed to estimate the volume of brain tissue and multiplied by the scaling factor.

To assess GM loss on a voxel-level between groups, a voxel-based morphometry (VBM) analysis was performed [23], [24]. The individual GM probability maps were registered to standard space using non-linear registration [25]. These images were averaged and flipped along the x-axis to create a symmetric, study-specific GM-template. All native GM images were non-linearly registered to the template and “modulated” to correct for local expansion/contraction. The modulated GM-density maps were smoothed with an isotropic Gaussian kernel (sigma 3 mm). A non-parametric permutation test (randomise, FSL; 5000 permutations) was used to assess between-group differences on a voxel-level. Family-wise error (FWE) correction for multiple comparisons across space was performed, implementing threshold-free cluster enhancement (TFCE) at p<0.05 [26].

### RSNs functional connectivity

**Figure 1:**
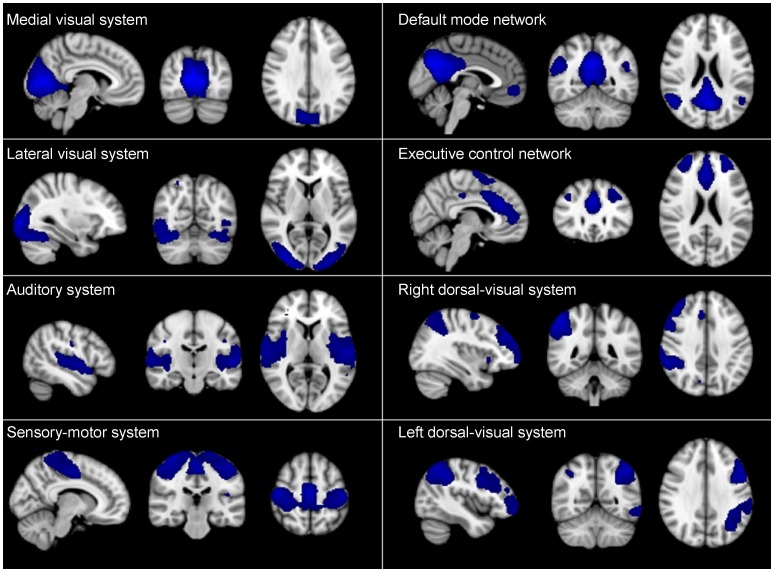
Resting state network maps. Standard masks of the eight resting state networks (RSNs) as described in Beckmann et al. (2005) are freely available and were downloaded from http://www.fmrib.ox.ac.uk/analysis/royalsoc8/. Blue regions show brain regions included in the RSNs; the medial-visual system, the lateral-visual, auditory system, sensori-motor system, the default mode network, the executive control network, right dorsal visual system and left dorsal visual system.

Default FSL-settings were used unless otherwise specified. In short, individual rs-fMRI data underwent motion correction, spatial smoothing using a 5 mm full-width-at-half-maximum (FWHM) Gaussian kernel, high-pass temporal filtering (0.01 Hz), removal of non-brain tissue, and registered to standard space (MNI152) via the T1-weighted image; using non-linear registration with a warp resolution of 10 mm. The warp resolution refers to the spacing between control points, and thus the smoothness, of the deformation field. In order to identify individual functional connectivity maps, a dual-regression technique was used [27] (http://fsl.fmrib.ox.ac.uk/fsl/fslwiki/DualRegression; File S1), using standard masks of RSNs [16] as input. These standard masks are freely available and include eight RSNs (Figure 1); the medial-visual system, lateral-visual system, auditory system, sensory-motor system, default mode network (DMN), executive control network, left and right dorsal visual system. In addition, two seed-regions in white matter and CSF were included to correct for physiological noise. The RSN masks and both seeds were resampled at 4 mm isotropic. First, spatial masks of the standard RSNs were used in a linear spatial regression against the individual rs-fMRI data, in order to find time signals for each subject associated with each RSN-mask. Second, these individual matrices were used in a temporal regression against the associated rs-fMRI data set to estimate subject specific spatial maps [27]–[29]. A non-parametric permutation test (5000 permutations) was used to assess interaction effects of diagnosis (AD vs. controls) with age (“young” <65 years vs. “old” ≥65 years) and between-group differences on a voxel-level within the boundaries of the RSN-masks. Voxel-wise GM probability maps (resampled at 4 mm) and gender were included as covariates. FWE correction for multiple comparisons across space was performed, implementing TFCE at p<0.05. Since 8 relevant RSNs were examined for 4 groups, a correction for the number of tests was included, resulting in p<0.0016 being significant (8 RSNs×4 groups; 0.05/32). Regions that showed differences between early-onset and late-onset AD were used to extract mean functional connectivity values (mean z-scores) for each patient, and used in a correlation analysis with cognition.

### Spatial overlap of atrophy with functional connectivity differences

To quantitatively evaluate the spatial overlap between atrophy and functional connectivity differences, the Dice Similarity Coefficient (DSC) was calculated. The DSC measures overlap between two segmentations (A and B), and is defined as DSC(A,B) = 2(A∩B)/(A+B) where ∩ is the intersection [30]. A DSC of 0 represents no overlap and 1 represents perfect overlap.

### Neuropsychological tests

Test scores that were not normally distributed were log-transformed. z-scores were calculated for all test-scores. The inverse (−1*zscore) of all tests measuring time were calculated, so that a lower score consistently represented poorer performance. Five cognitive domains were defined based on averaged z-scores; the memory domain included Visual Association Test (VAT) and immediate and delayed recall of the Dutch version of the Rey Auditory Verbal Learning Task (RAVLT). The attention domain included Trail Making Test (TMT) part A and Digit Span forward. The language domain included VAT naming and category fluency. Executive functioning included TMT part B and Digit Span backward. Visuo-constructive functioning consisted only of the Rey Complex Figure Copy test. For a detailed description of neuropsychological tests see Smits et al. [4]. For 46 out of the 48 AD patients neuropsychological data were available (19 early-onset and 27 late-onset AD patients). For these 46 AD patients, not all cognitive domains were tested, resulting in a different number of subjects’ data available per cognitive domain. For the 5 cognitive domains, age and MMSE score did not differ significantly for AD patients with data available compared to AD patients with no cognitive data available.

### Non-imaging statistics

Differences between groups were assessed using an Independent Samples T-test, a Mann-Whitney U test or a Chi-square test where appropriate. In order to test the effect of age on functional connectivity in more detail (in a non voxel-wise approach), a linear regression analysis was performed with an interaction term between diagnosis (AD vs. controls) and age (“young” <65 years vs. “old” >65 years) on mean functional connectivity (mean z-scores within the RSNs mask), accounting for gender and GM volume. A two-tailed Spearman correlation was used to assess the associations between mean RSNs functional connectivity (z-scores) with neuropsychological test score (z-scores) across AD patients. Second, in order to examine these associations in early-onset and late-onset AD groups separately, a linear regression analysis with an interaction term between age of onset (early-onset AD vs. late-onset AD) and functional connectivity on the relationship with cognitive performance was examined. When the interaction term with age of onset was significant, Spearman correlations were performed for that association within both early-onset and late-onset AD patients separately. A p-value below 0.05 was considered significant. All statistical analyses were performed using SPSS (version 20.0; SPSS, Chicago, IL, USA).

## Results

Based on age at diagnosis, our sample contained 20 early-onset and 28 late-onset AD patients. Data of 15 “young” (<65 years old) and 31 “old” (≥65 years old) age-matched controls were included. As expected, MMSE score and NGMV were found to be significantly lower in AD patients when compared to controls (Table 1). The voxel-wise interaction analysis yielded no significant results, which was probably due to low power. However, when examining mean functional connectivity for the RSNs, significant interactions of diagnosis with age were found for all 8 RSNs.

**Table 1:** Subject characteristics for early-onset Alzheimer’s disease (AD) patients (disease onset <65 years), late-onset AD patients (disease onset ≥65 years), young (<65 years) - and old (≥65 years) healthy age-matched controls (HC).

	Early-onset AD	Late-onset AD	Young HC	Old HC
N	20	28	15	31
Age at time of scan	59±2.4	72±4.9	61±2.8	72±4.3
Disease duration at time of scan (years)	1.2±1.2	1.2±0.9	n.a.	n.a.
Gender m/f	14/6	17/11	9/6	17/14
MMSE score	22.7±2.8† ^,^ ‡	22.0±2.8† ^,^ ‡	29±1.1♦ ^,^ ¶	29±1.1♦ ^,^ ¶
NGMV (L)	0.72±0.07† ^,^ ‡	0.75±0.06† ^,^ ‡	0.88±0.05♦ ^,^ ¶	0.80±0.05♦ ^,^ ¶
Education	5.33±1.08	5.19±1.30	5.89±1.27	5.71±1.30
Relative displacement (mm)	0.09±0.07	0.12±0.07	0.09±0.04	0.11±0.06
Using AD medication (%)*	65	64	0	0

Data are presented as means ± standard deviations.

Mini Mental State Examination (MMSE) score and Normalized Gray Matter Volume (NGMV) in litre (L). Level of education using Verhage’s classification [44]. Not applicable (n.a.). Alzheimer’s disease (AD). Healthy controls (HC).

*All AD patients that were on AD medication used Reminyl, with exception of 1 early-onset AD patient, who used Exelon. Both are competitive and reversible cholinesterase inhibitors.

♦ significantly different from early-onset AD.

¶ significantly different from late-onset AD.

† significantly different from young controls.

‡ significantly different from old controls.

### Effect of aging on functional connectivity

Functional connectivity was not different in any of the RSNs when comparing the two control groups (young vs. old controls). This implies that there is no general effect of aging on functional connectivity.

### Functional connectivity differences AD patients and age-matched controls

**Figure 2:**
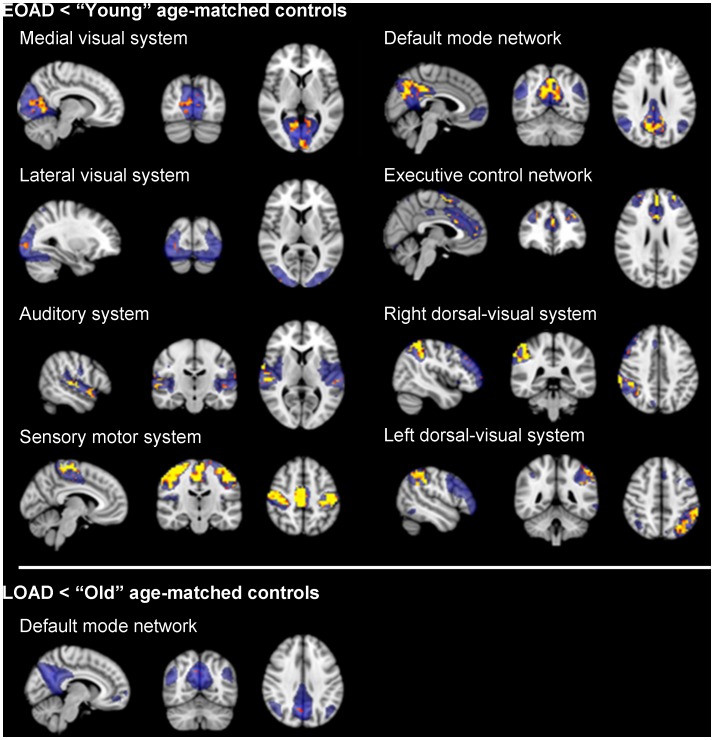
Lowered functional connectivity in AD patients when compared to age-matched controls. Upper panel shows lower functional connectivity in early-onset AD patients when compared to ‘young’ age-matched controls. Lower panel shows decreased functional connectivity in late-onset AD patients (EOAD) when compared to ‘old’ age-matched controls. Standard maps of the Resting State Networks (RSNs) are shown in transparent blue. Upper panel: Lower functional connectivity was found in early-onset AD patients when compared to aged-matched young controls within the medial-visual system, lateral-visual system, auditory system, sensory-motor system, default mode network, the executive control network and bilateral dorsal-visual stream. Lower panel: late-onset AD patients (LOAD) show lower functional connectivity when compared to old age-matched controls within the default mode network only. Results are displayed in radiological orientation on standard MNI space (MNI152 2 mm), after correction for multiple comparisons across space (p<0.05). Gender and voxel-wise gray matter maps were used as covariates. Brighter colors represent most significant results. Abbreviations: R = right. L = left.

Early-onset AD patients showed lower functional connectivity when compared to “young” age-matched controls in all 8 RSNs (Figure 2; upper panel). For the medial-visual system, lower functional connectivity was seen in precuneus and occipital cortex. For the lateral-visual cortex, this was apparent in bilateral occipital cortex. For the auditory system, superior temporal regions and Heschl’s gyrus showed lower functional connectivity. For the sensory-motor system, lower functional connectivity was seen in pre- and postcentral gyrus. For the DMN, lower functional connectivity was seen within the precuneus, posterior cingulate and medial frontal cortex. For the executive control network this was seen in anterior cingulate, paracingulate and superior frontal gyrus. Finally, for the bilateral dorsal-ventral system, lower functional connectivity in early-onset AD patients compared to age-matched controls was seen in bilateral anterior supramarginal gyrus, occipital and middle frontal gyrus. When examining late-onset AD patients compared to age-matched controls, lower functional connectivity for AD patients was found only within the DMN and included a small part of the precuneus (Figure 2; lower panel). When correcting for the number of tests performed, significant results remained, although slightly smaller. For peak-coordinates and number of voxels see Table 2.

**Table 2:** MNI-coordinates (MNI 152, 2 mm) of peak voxel and total number of voxels that showed significant lower functional connectivity within the resting-state networks (RSNs) when comparing early-onset AD (EOAD) patients with age-matched young healthy controls (HC), when comparing late-onset AD (LOAD) patients with age-matched old HC and in a direct comparison between early-onset AD and late-onset AD patients.

Resting State Network	MNI-coordinates ofpeak (x,y,z)	Number of voxels (FWEcorrected p<0.05)	Number of voxels (FWEcorrected p<0.0016)
**EOAD<Young HC**			
Medial-visual system	18,−62,0	3386	221
Lateral-visual system	34,−94,4	1365	9
Auditory system	66,−14,−4	2201	369
Sensory-motor system	62,−10,20	6668	3680
Default mode network	18,−62,20	3990	1350
Executive control network	2,46,4	2670	1101
Right dorsal visual system	42,−42,32	2231	864
Left dorsal visual system	−46,−38,36	1663	438
**LOAD<Old HC**			
Default mode network	10,−62,36	14	0
**EOAD<LOAD**			
Auditory system	50,−26,8	35	0
Sensory-motor system	34,−22,48	441	0
Default mode network	18,−62,20	210	0
Right dorsal visual system	54,14,28	19	0
Left dorsal visual system	−42,−66,48	114	0

Number of voxels are reported when using the FWE corrected p<0.05 and when correcting also for the number of tests (8 RSNs×4 groups) resulting in FWE corrected p<0.0016.

### Functional connectivity differences early-onset and late-onset AD patients

**Figure 3:**
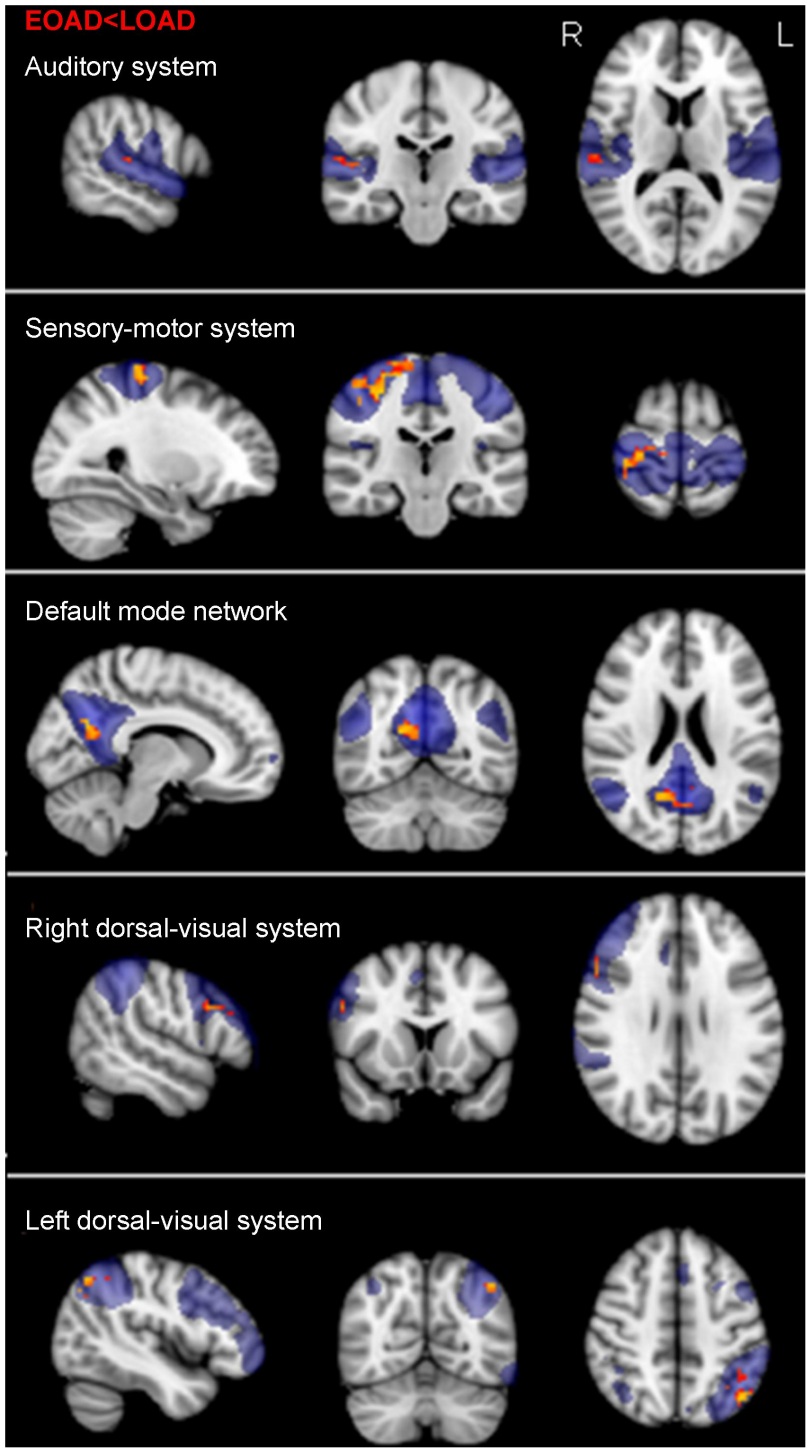
Functional connectivity in early-onset AD patients compared to late-onset AD patients. Standard maps of the Resting State Networks (RSNs) are shown in transparent blue. Lower functional connectivity was found in early-onset AD (EOAD) patients when compared to late-onset AD (LOAD) patients within the default mode network, the auditory system, the sensory-motor system, and bilateral dorsal visual system. Results are displayed in radiological orientation on standard MNI space (MNI152 2 mm), after correction for multiple comparisons (p<0.05). Gender and voxel-wise gray matter maps were used as covariates. Brighter colors represent most significant results. Abbreviations: R = right. L = left.

Lower functional connectivity was found in 5 of the 8 RSNs in early-onset AD when compared to late-onset AD patients (Figure 3): within the auditory-, sensory-motor-, bilateral dorsal visual system and the DMN. Areas with lower functional connectivity for early-onset AD were similar to the comparison with the control group, though smaller. For the auditory system lower functional connectivity was found in the right superior temporal gyrus and Heschl’s gyrus. For the sensory-motor system, lower functional connectivity was seen in bilateral precentral/postcentral gyrus and the precuneus. Lower functional connectivity in the DMN was observed in the posterior cingulate cortex and right lateral parietal cortex. For the right dorsal-visual system, lower functional connectivity was seen in the right angular-, middle frontal gyrus and occipital cortex. Finally, for the left dorsal-visual system, lower functional connectivity in early-onset AD was found in the left angular gyrus and lateral occipital cortex. When correcting for the number of tests performed, no significant results remained. No significant differences between early-onset and late-onset AD were found in the medial- and lateral visual system, and the executive control network. No area in any of the RSNs was identified with lower functional connectivity for late-onset AD compared to early-onset AD patients.

### Only partial spatial overlap functional connectivity and atrophy

**Figure 4:**
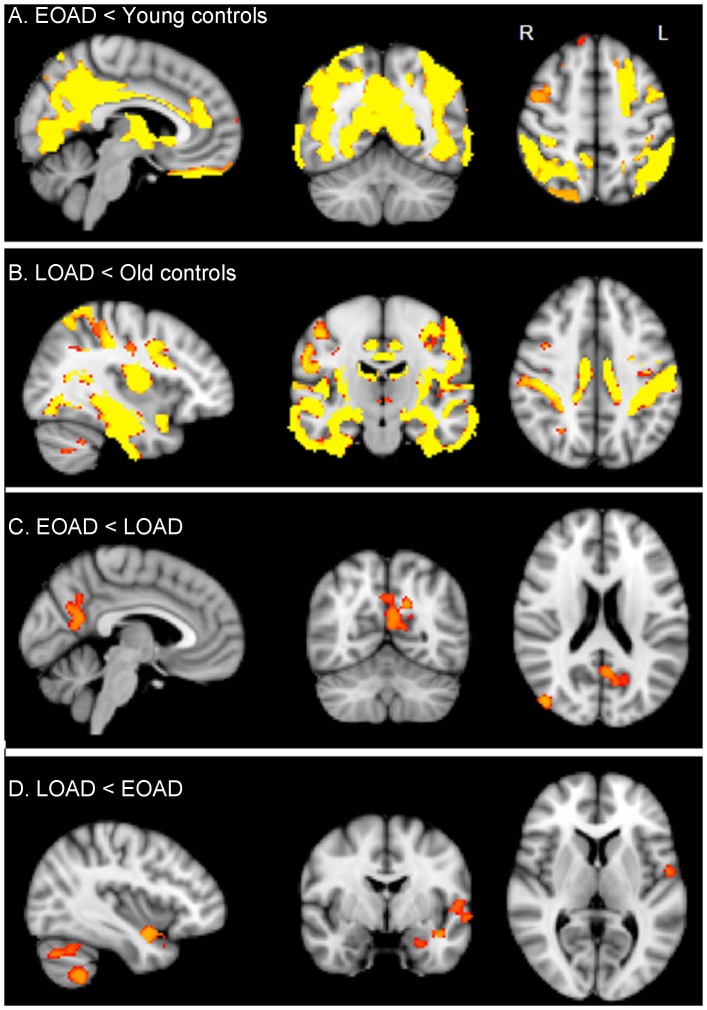
Gray matter loss. Regions of decreased gray matter volume in early-onset AD (EOAD) patients when compared to age-matched young controls (A). Regions of decreased gray matter volume in late-onset AD (LOAD) patients when compared to age-matched old controls (B). Regions of decreased gray matter volume in early-onset AD patients when compared to late-onset AD (C). Regions of decreased gray matter volume in late-onset AD patients when compared to early-onset AD (D). Results are corrected for multiple comparisons (p<0.05) and are shown in radiological orientation on standard MNI space (MNI152 2 mm). Brighter colors represent most significant results. R = Right. L = Left.

Lower GM volume in early-onset AD patients compared to age-matched controls was seen in the precuneus, posterior cingulate cortex, bilateral parietal cortex, medial inferior occipital cortex and anterior cingulate cortex (Figure 4A). DSC for lower functional connectivity and GM loss in early-onset AD compared to controls was 0.22. When comparing late-onset AD patients to age-matched controls, lower GM volume was seen in medial, lateral and superior temporal cortex, insular cortex, small parts of the anterior and posterior cingulate gyrus, bilateral postcentral gyrus, inferior occipital cortex and small regions in cerebellum (Figure 4B). Lower functional connectivity did not overlap with atrophy for late-onset AD patients compared to controls. Lower GM volume in early-onset AD patients compared to late-onset AD patients was seen in parts of the precuneus, posterior cingulate cortex and right lateral parietal cortex (Figure 4C). Overlap of lower functional connectivity with GM loss in early-onset AD patients compared to late-onset AD resulted in a DSC of 0.12. Late-onset AD patients showed lower GM volume when compared to early-onset AD patients in small regions of the left temporal cortex: left planum polare, parahippocampal gyrus, insular cortex and precentral gyrus. Lower GM volume in late-onset AD patients than early-onset AD was also observed in the cerebellum (Figure 4D). Lower functional connectivity for late-onset AD patients compared to early-onset AD patients was not observed and therefore did not overlap with atrophy.

### Neuropsychological performance

Table 3 lists performance on raw neuropsychological tests and the five cognitive domains for early-onset and late-onset AD patients. Regarding the five cognitive domains, there were no significant differences between early-onset and late-onset AD patients. Although not significant, early-onset AD patients did tended to have lower scores on visuo-constructive, executive functioning and attention and late-onset AD patients lower scores on memory and language.

**Table 3:** Neuropsychological test performance of early-onset and late-onset Alzheimer’s disease (AD) patients.

	Early-onset AD		Late-onset AD		
Raw data	N		N		p-value
Memory					
VAT	17	4.88±4.48	25	4.00±3.43	0.47
RAVLT, total immediate recall	15	24.73±6.03	27	19.63±8.17	**0.04**
RAVLT, delayed recall	15	1.93±2.40	27	0.74±1.48	0.16
Language					
VAT naming	15	11.20±1.66	24	10.67±2.24	0.43
Category fluency	14	11.79±4.06	25	11.52±4.39	0.85
Visuo-constructive functioning					
Rey-figure, copy	9	24.33±7.22	21	27.12±8.43	0.40
Executive functioning					
TMT B*	13	387.54±347.82	22	280.27±212.87	0.26
Digit Span backward	18	6.11±2.17	26	7.69±3.27	0.08
Attention					
TMT A*	17	100.71±79.56	25	71.40±25.74	0.09
Digit Span forward	18	11.11±2.63	26	11.62±3.13	0.58
**Composite scores (mean z-scores)**	**Early-onset AD**		**Late-onset AD**		**p-value**
Memory	0.32±1.05		−0.18±0.68		0.09
Language	0.05±0.77		−0.07±0.83		0.69
Visuo-constructive functioning	−0.17±0.86		0.07±1.07		0.56
Executive functioning	−0.15±0.90		0.17±0.83		0.30
Attention	−0.22±0.79		0.10±0.60		0.15

Upper part; raw scores on the different neuropsychological tests. Lower part; mean z-scores for the composite cognitive domains.

Group differences were assessed with an independent samples t-test.

*higher values represent poorer performance.

### Association functional connectivity RSNs with cognition in AD

**Figure 5:**
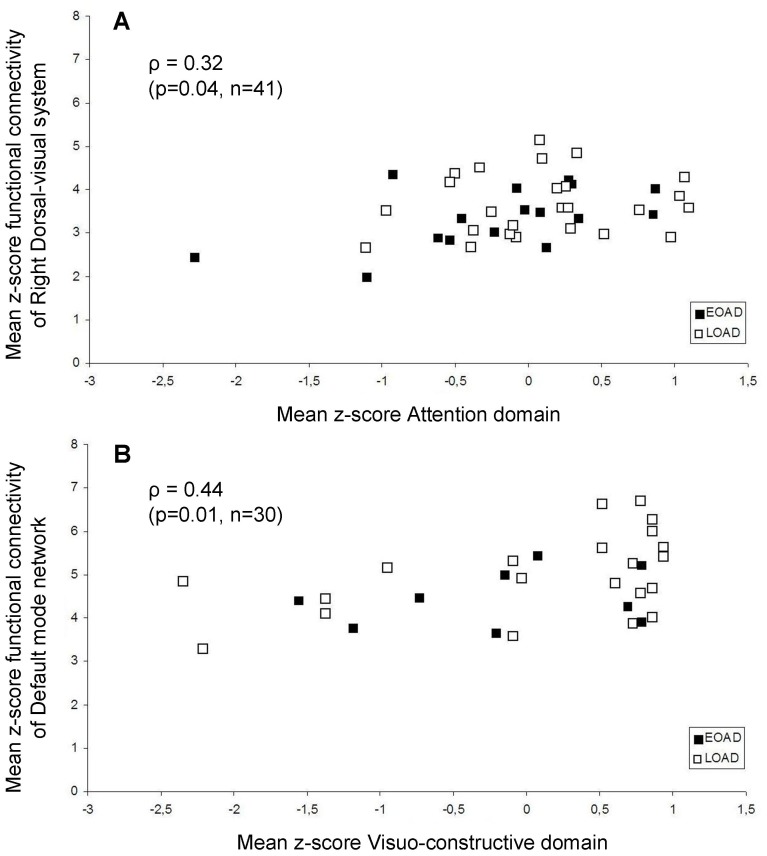
Significant correlations of functional connectivity with cognition in AD patients. Scatterplots of significant Spearman correlations across AD patients are shown; mean z-scores representing functional connectivity within the resting state network (RSNs) are displayed on the y-axis. Mean z-scores composing the cognitive domains are displayed on the x-axis. Functional connectivity of the right dorsal visual stream (A) was associated with attention. Functional connectivity of the default mode network (B) was associated with visuo-construction. Black squares represent early-onset (EOAD), white squares represent late-onset AD patients (LOAD). Spearman correlation coefficients (ρ) are reported, with corresponding p-value and number of subjects included in the correlation analysis.

Since we observed no differences in cognitive performance between early-onset and late-onset AD, correlations with functional connectivity across patients were calculated (Table 4). Of the 25 possible correlations, 2 correlations were significant; Lower functional connectivity of the right dorsal-visual system (ρ = 0.32, p = 0.04) was associated with poorer attention across all patients. Lower functional connectivity of the DMN (ρ = 0.44, p = 0.01) was associated with poorer visuoconstructive performance across all AD patients. For scatterplots of significant correlations across patients see Figure 5. Next, the interaction term between age of onset (early-onset AD vs. late-onset AD) and functional connectivity on the relationship with cognitive performance was examined. This interaction term was found significant for the relationship between functional connectivity of the sensory-motor system and memory performance. When exploring this association in more detail it was found that higher functional connectivity of the pre/post central gyrus within the sensory-motor system was associated with poorer memory in late-onset AD patients (ρ = −0.47, p = 0.02).

**Table 4:** Spearman correlation coefficients of functional connectivity (mean z-score) within significant clusters within the resting-state network (RSNs) maps with performance on 5 cognitive domains (mean z-score).

	Auditorysystem	Sensory-motorsystem	Default ModeNetwork	Right dorsalvisual system	Left dorsalvisual system
**Memory (n = 38)**	0.07 (0.67)	−0.23 (0.16)	−0.04 (0.84)	−0.17 (0.30)	−0.03 (0.86)
**Language (n = 36)**	0.16 (0.37)	0.09 (0.59)	0.28 (0.10)	0.13 (0.45)	0.05 (0.76)
**Executive (n = 34)**	0.03 (0.88)	0.03 (0.88)	0.22 (0.21)	0.14 (0.43)	0.002 (0.99)
**Visuo-construction (n = 30)**	0.28 (0.13)	0.04 (0.83)	0.44* (0.01)	0.24 (0.20)	0.23 (0.23)
**Attention (n = 41)**	0.22 (0.17)	0.31 (0.05)	0.30 (0.05)	0.32* (0.04)	0.19 (0.22)

2-tailed Spearman correlation coefficients are reported (p-value) *p<0.05. Significant correlations are highlighted in bold.

## Discussion

The main finding of this study is that functional connectivity within several RSNs was lower in early-onset AD compared to late-onset AD patients. Lower functional connectivity was found in parietal, occipital and frontal regions that have previously been associated with early-onset AD, but also in sensory-motor and auditory cortex. These widespread alterations in functional connectivity might explain cognitive problems seen in early-onset AD patients.

### Lower functional connectivity in early-onset AD in the DMN and dorsal-visual system

Most studies have associated DMN disruptions with AD, especially within the precuneus [18], [31], typically focussing on late-onset AD. This was confirmed in this study; lower functional connectivity of the DMN was found for late-onset AD patients when compared to controls. We now also show implications of the DMN for different subtypes within AD. Functional connectivity of the DMN was found to be more severely affected in early-onset compared to late-onset AD within the parietal cortex, which was only partly attributable to atrophy. The results of our VBM analysis were in line with literature; early-onset AD patients showed more cortical atrophy in posterior areas [12], [13], where late-onset AD patients had more pronounced (left) medial temporal lobe and cerebellar atrophy, which is in agreement with Möller et al. [32]. Indeed, it is already known that the precuneus is most vulnerable in early-onset AD with pronounced hypometabolism [6], [7], [9] and atrophy [12], [13]. Posterior cortical atrophy has been reported to be associated with impaired processing of visual-spatial information [33], a feature of early-onset AD [4], [34]. This was confirmed by our results; visuo-construction was associated with functional connectivity of the precuneus within the DMN. Similar to the present study, Gour and colleagues investigated functional connectivity in early-onset and late-onset AD patients using rs-fMRI [17]. To our knowledge this is the only other study examining the direct comparison between early-onset and late-onset AD patients using rs-fMRI. In contrast to our findings, Gour et al. did not find differences within the DMN in a direct comparison between early-onset and late-onset AD patients. They did show that both early-onset and late-onset AD patients both showed lower functional connectivity within the DMN when compared to age-matched controls, but found this in frontal regions, where we found this in posterior cingulate cortex. The conflicting results are difficult to explain, but could be due to the different techniques used to establish functional connectivity, which is discussed below.

In the present study, lower functional connectivity within both left and right dorsal-visual system was seen in early-onset compared to late-onset AD patients. For the right dorsal-visual system this was observed in right angular-, middle frontal gyrus and occipital cortex. For the left dorsal-visual system the same regions were found, with the exception of the middle frontal gyrus. The occipital cortex, besides posterior regions, has also been reported to show more severe hypometabolism [7] and reduced brain activity measured with EEG [11] in early-onset AD. Interestingly, hypometabolism of the (left) middle frontal gyrus have been found associated with poorer performance on the executive system in working memory in early-onset AD [8]. In this study we found that functional connectivity of the right dorsal-visual system was associated with performance on cognitive tasks involving attention. The dorsal-visual system is supposedly involved in spatial awareness and guidance of actions [35]. Similar to our results, Gour et al. [17] found lower functional connectivity in early-onset AD patients within the dorso-lateral prefrontal network compared to late-onset AD, which was observed in the anterior cingulate, precentral gyrus, inferior and superior frontal cortex. In addition, Gour et al. also identified the cuneus, precuneus and inferior parietal cortex, which was not replicated by the present study.

### Lower functional connectivity in early-onset AD in auditory and sensory-motor system

Lower functional connectivity in early-onset AD patients included areas that are vulnerable in these patients [7], [9] but also extended well beyond these regions. This is in line with the hypothesis that functional changes precede atrophy [36]. Functional connectivity measures functional integrity of spatially distinct brain regions [15]. Local disturbances could therefore have widespread effects. Within the auditory system, lower functional connectivity in early-onset AD was found within right Heschl’s gyrus, which processes auditory stimuli and is involved in semantic tasks [37]. This might explain why early-onset AD patients present more often with language problems than late-onset AD patients [5]. Functional connectivity of the pre/postcentral gyrus was affected in early-onset AD within the sensory-motor system. Infarctions in the (left) supplementary motor area have been related to apraxia [38], which could explain why early-onset AD patients present more frequently with apraxia [3]. Unfortunately, no behavioral data assessing motor planning was available for this study.

### Functional connectivity less affected in late-onset AD

We hypothesized that late-onset AD patients would show lower functional connectivity in regions linked to memory performance, since memory problems are most prominent in late-onset AD [2]. In addition, Gour et al. [17] found lowered functional connectivity in the anterior-temporal network (ATN) in late-onset AD patients. The ATN is believed to be involved in declarative memory [39]. However, our RSN analysis did not identify areas which were more severely affected in late-onset AD when compared to early-onset AD patients. This finding is difficult to interpret, especially with respect to recent EEG-findings, where it was shown that late-onset AD patients show more disturbed brain activity in temporal regions [11]. Interestingly, it was found that for late-onset AD patients higher functional connectivity of the pre/post central gyrus within the sensory-motor system was associated with poorer memory. This relationship might be somewhat counterintuitive since lower functional connectivity would be expected to be related to poorer performance on cognitive tasks. Also, sensorimotor regions are found to be relatively spared in AD [40] and not directly involved in memory performance. Agosta et al. [41] performed a functional connectivity analysis of the sensorimotor system in controls, MCI and AD patients. One of their findings was that functional connectivity of the left sensorimotor cortex with the cingulum was increased in AD patients compared to MCI patients and controls. The authors speculated that this reflected a disrupted between-network connectivity in AD patients, possibly reflecting progressively impaired deactivation [41] or compensatory mechanisms.

### RSNs analysis vs. seed-based analysis

Seed-based connectivity analyses are a traditional way to compare resting-state networks in fMRI [42] connected to specific seed regions. We chose to use the dual regression method to test multiple possible resting state networks, as we did not use prior knowledge for choosing a seed region. With multiple seed-based analyses, these networks would be tested independently, whereas dual regression provides an integrated analysis of the networks and their interactions [43].

### Limitations and recommendations

Some limitations of this study should be taken into consideration. Our between group analysis was limited to the boundaries of the RSNs. Examining extended effects of AD on functional connectivity with areas outside the RSNs are of future interest, possibly by using a whole-brain graph analytical measure. Second, after correcting for the number of tests performed for the RSNs and number of groups, results for the direct comparison between early-onset and late-onset AD patients were not significant anymore. Not all subjects were able to perform or complete all neuropsychological tests, which may have resulted in an under-estimation of neuropsychological abnormalities in more severely affected patients, although age and MMSE score was not significantly lower in patients that did not have cognitive data available. Also, cognitive data was not available for healthy controls. The limited number of subjects with cognitive data available could have resulted in low statistical power for the correlation analyses and should therefore interpreted with caution. Also, the association of functional connectivity with cognitive performance should be interpreted with caution, since this is not likely to be a one-to-one relationship. Between RSNs connectivity could further elucidate cognitive performance, since networks are likely to work together.

## Supporting Information

File S1 (PDF)
