# Supplementary material for: Widespread Disruption of Functional Brain Organization in Early-Onset Alzheimer’s Disease
Source: PLoS One. 2014 Jul 31;9(7):e102995. doi: 10.1371/journal.pone.0102995 (PMC4117463; doi:10.1371/journal.pone.0102995)
Supplement: File S1 — (PDF) [file pone.0102995.s001.pdf]

Imperial College London

FMRIB Oxford Centre for Functional MRI of the Brain

Group comparison of resting-state FMRI data using multi-subject ICA and dual regression

Christian F. Beckmann[1,2], Clare E. Mackay[2], Nicola Filippini[2] and Stephen M. Smith[2]

[1]Division of Neuroscience and Mental Health, Hammersmith campus, Faculty of Medicine, Imperial College London, UK

[2]FMRIB Centre, Department of Clinical Neurology, University of Oxford, UK

Introduction

Studies of resting FMRI data increasingly concern estimation at the group level, i.e. of differences in functional connectivity patterns between different subject groups and/or between data obtained under different conditions such as under pharmacological interventions. Seed-voxel/region-based regression approaches (e.g. [5]) and Independent Component Analysis (ICA) based techniques (e.g. [1,2]) have been used extensively in order to identify such patterns of functional connectivity under rest. While these approaches have proven useful in characterising these resting fluctuations at the individual subject level, extensions to group-level comparisons deserve continued investigation. In this work we discuss an analysis approach which combines multi-subject ICA with a dual regression approach in order to estimate such differences in functional connectivity in a principled way.

Method

Initial FMRI data pre-processing involves correction for head motion, spatial and temporal filtering (and potentially time-series normalization to unit variance) as well as co-registration of data to a common atlas space. The between-subject (group analysis) of the resting data then proceeds in three stages:

① *concat-ICA:* When analysing multiple subjects (or sessions) one can either form a 3D Space*Time*Subjects data tensor and use tensor-ICA [2], or concatenate all datasets temporally to form a 2D Space*ConcatenatedTime data matrix, and use concat-ICA (as in [1]). Concat-ICA is particularly useful when the effects of interest are not expected to have similar timecourses in all subjects' datasets - for example when looking for resting-state networks (RSNs). Multiple FMRI data sets are concatenated temporally and ICA is applied in order to identify *large-scale patterns of functional connectivity* in the population:

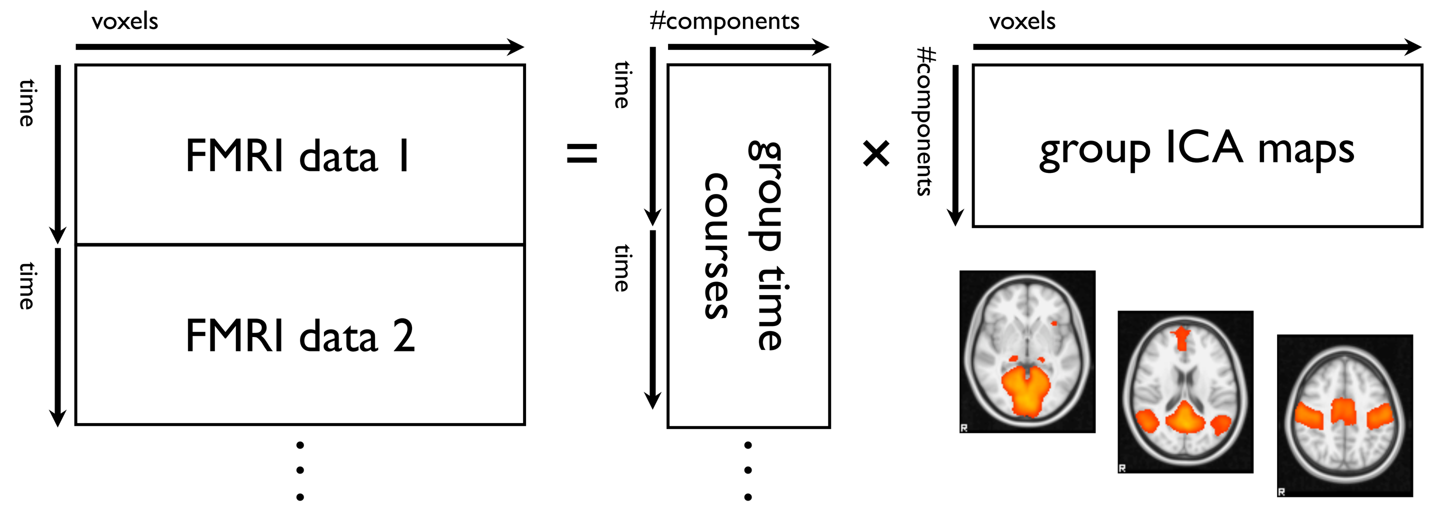

② *Dual Regression:* This is used to identify, within each of the *N* individual subjects' FMRI data, spatial maps and associated timecourses corresponding to the multi-subject ICA components. For each individual dataset separately:

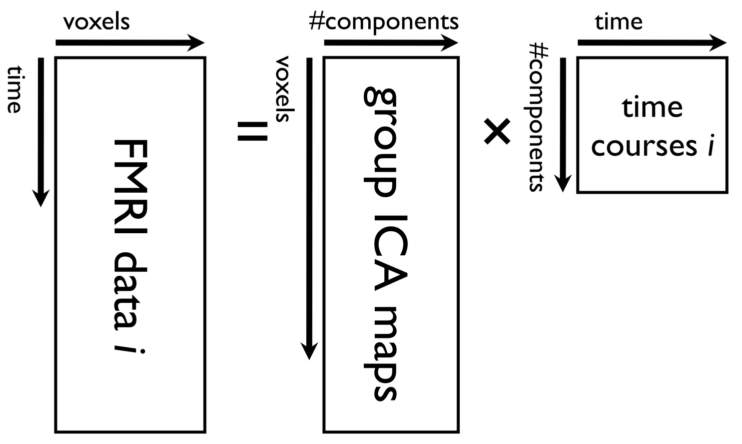

(A) use the group-level spatial maps as a set of spatial regressors in a GLM, to find temporal dynamics associated with each group-level map

(B) normalization of these timecourses to unit variance (optional, depending on what question the experimenter wants to ask later)

(C) use these timecourses as a set of temporal regressors in a GLM, to find subject-specific maps (still associated with the group-level spatial maps:

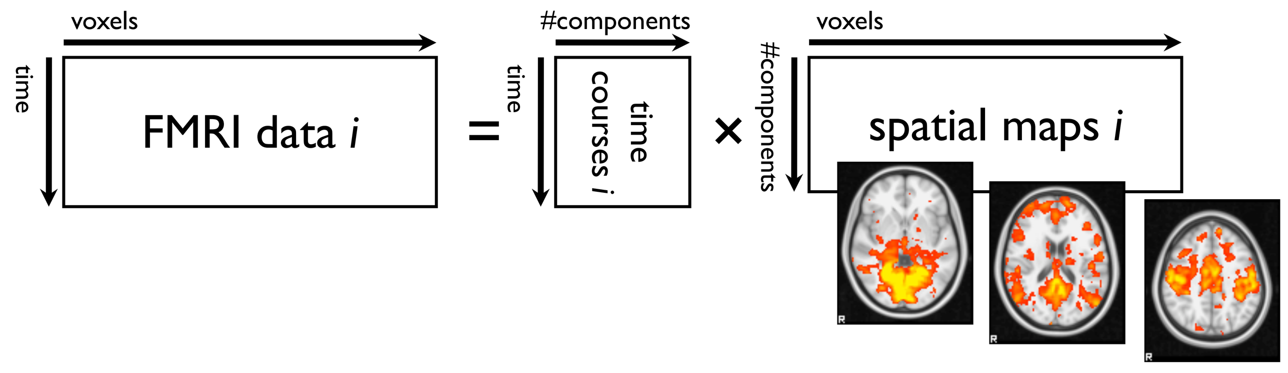

This results in pairs of estimates which form a dual space and jointly best approximate the original group ICA maps.

③ *Inference:* Finally, the different sets of spatial maps are collected across subjects into single 4D files (one per group-level ICA map) and analysed using non-parametric methods, e.g. permutation testing. This results in spatial maps characterising the between-subject group-consistency and/or between-subject group-differences:

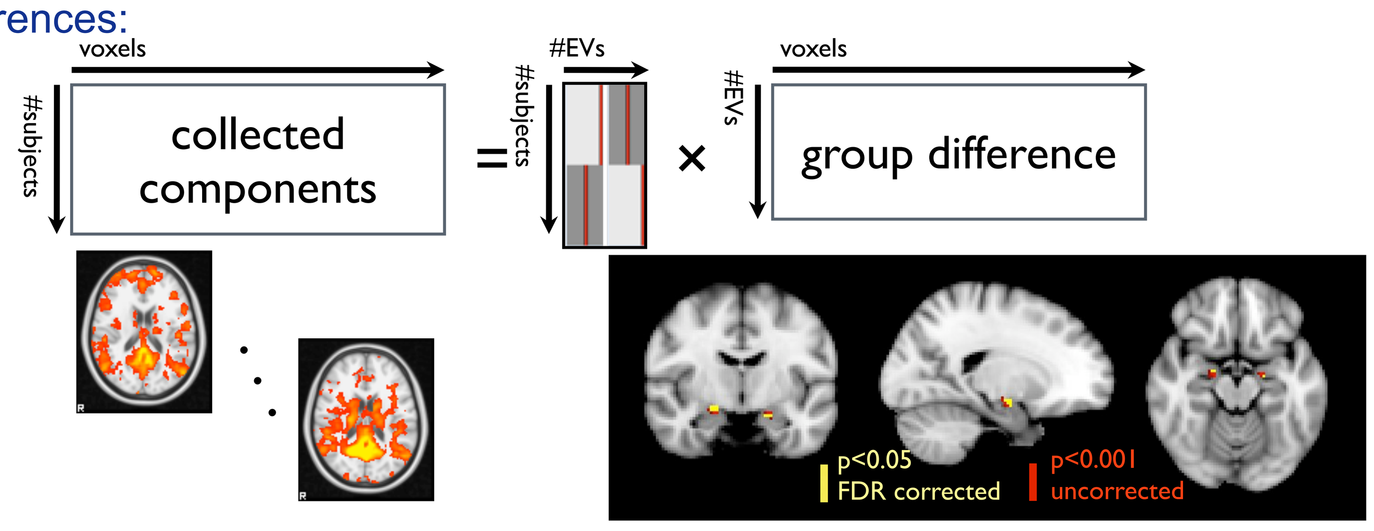

The example above illustrates this approach in a population of 36 healthy subjects (18 APOE-ε4 carriers and 18 matched controls, see [6] and #181 SU-AM for details). The initial group-ICA (①) identified multiple resting-state networks at the group level. By means of dual regression (②), corresponding subject-specific maps are being estimated which, when compared between groups using non-parametric permutation testing (③), reveal significantly increased hippocampal involvement in the 'default mode network' in ε4-carriers relative to non-carriers.

Dual Regression in context

The dual regression approach addresses a set of issues associated with alternative approaches for group resting-FMRI analysis:

- In contrast to a *seed-voxel or seed-region based analysis* approach the method described here does not rely on a single seed location but integrates the temporal information in the FMRI data across *multiple distributed networks* identified in the initial group ICA. The component maps of the initial ICA decomposition effectively define regions or networks of interest which are demonstrably relevant at the group level for the particular population (note, though, that instead of deriving the overall networks from the population under observation it is also possible to use pre-defined networks such as those described in [1], see #246 M-PM).
  Furthermore, this approach uses *multiple linear regression* instead of single regression and therefore can estimate significant differences in the presence of other structured effects in the data which otherwise might negatively impact on such comparisons. Due to the multiple linear regression framework it can also more directly address questions about the dynamic interaction between networks (see #78 F-PM, #186 F-PM, #246 M-PM)
- With respect to the alternative group-ICA methodology [4] the approach presented here estimates spatial and temporal dynamics at the subject level based on regression against the original data rather than estimating subject-specific maps by means of 'back-projection'. In the back-projection approach the estimated spatial maps necessarily lie within the space defined by (the pseudoinverse of) the initial subject-specific major Eigenspaces (PCA). As such, the final between-subject comparison (e.g. inference on the between-group difference) becomes dependent on the initial subject-specific reduction stages. While this is computationally efficient, the final statistical comparison on the back-projected maps can lead to significantly inaccuracies (both in terms of false-positive and false-negative detections). This is of particular importance as the effect differences can be small relative to the overall effect sizes.

Back-Projection vs. Dual Regression

Two simulated data sets, based on a set of 10 uncorrelated spatial maps and orthogonal (i.e. uncorrelated) time courses embedded in Gaussian random noise:

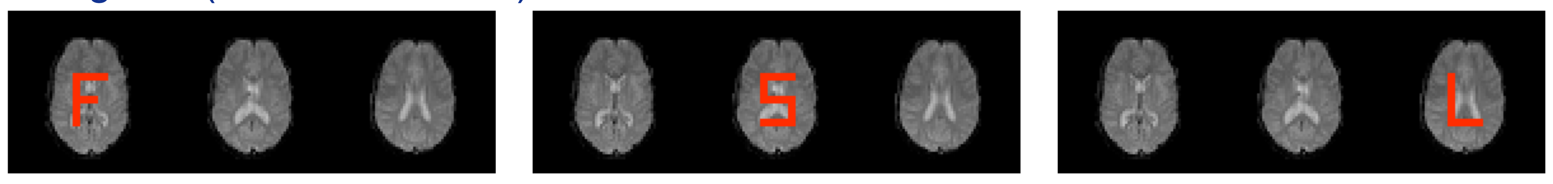

- group A: The three signals of interest (above) are amongst the 5 strongest signals.
- group B: Signals 'F' & 'S' remain unaltered, all other signals (including 'L') increase in strength by a factor of 2. As a consequence, signal 'S' no longer is amongst the 5 strongest components, whereas signals 'F' and 'L' remain within this set.

Data sets A and B are analysed using group-ICA as in [4] and concat-ICA with dual regression, reducing data into a 5-dimensional subspace. In both cases, the multi-subject ICA maps identify all 3 signals of interest. Following back-projection as in [4] and dual regression as described here we can illustrate the difference in terms of:

① *Sensitivity to the initial PCA-based dimensionally reduction:*
Signals 'F' and 'S' are both contained at equal strength in both data sets. Nevertheless, because 'S' is not represented in the initial reduced space of B, the back-projected signal 'S' differs between data sets A and B (i.e. detection of a significant difference for signal 'S'; *false-positive detection*).

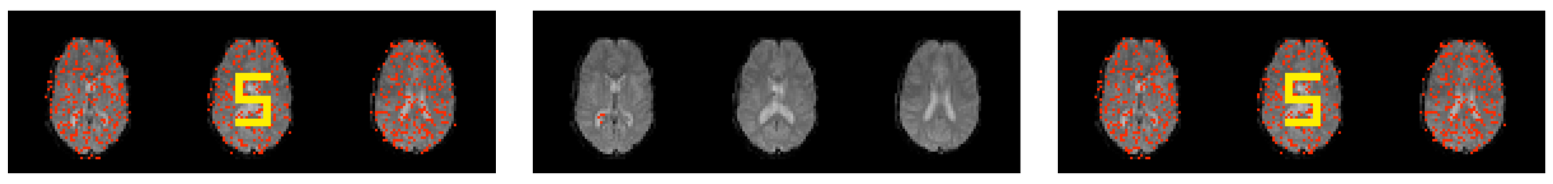

back-projected 'S' from A | back-projected 'S' from B | Difference map

By comparison, maps after dual regression are identical, i.e. correctly reflect that signals 'F' and 'S' are the same between data sets A and B (*true negative detection*)

② *Sensitivity to global amplitude differences:*
Signal 'L' is twice as strong in data set B compared to data set A.
Back-projected spatial maps for 'L' are identical (i.e. no detection of a significant difference between data sets A and B; *false negative detection*).
By comparison, the dual regression results (with time series normalisation) reflect differences in the global amplitude between the data sets (*true positive detection*).

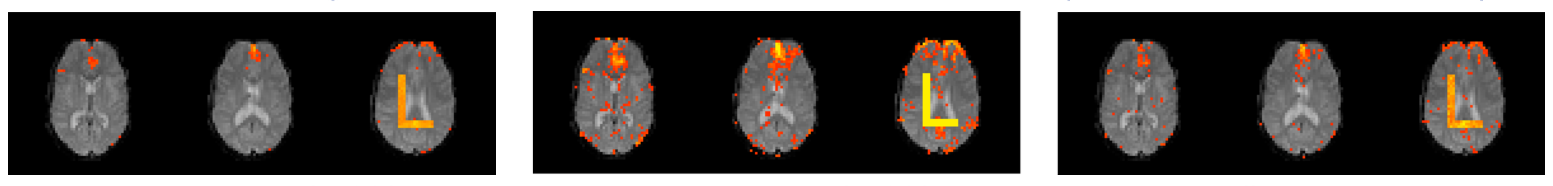

dual regression result for 'L' from A | dual regression result for 'L' from B | Difference map

Conclusion

We have described a principled analysis approach which permits the identification of *between-subject differences* in resting functional connectivity based on *between-subject similarities* using a dual regression approach within the framework of multi-subject-ICA analysis. All the analysis steps can be carried out using tools from the FMRIB Software Library (FSL [3], **http://www.fmrib.ox.ac.uk/fsl**)

REFERENCES

[1] Beckmann, CF et al.(2005), 'Investigations into resting-state connectivity using independent component analysis', Philosophical Transactions of the Royal Society, 360(1457):1001-1013.
[2] Beckmann, CF et al. (2005), 'Tensorial extensions of independent component analysis for multisubject FMRI analysis', Neuroimage, 25(1):294-311.
[3] Smith, SM et al. (2004), 'Advances in functional and structural MR image analysis and implementation as FSL', Neuroimage, 23(S1):208-219.
[4] Calhoun, VD et al. (2001), 'A Method for Making Group Inferences from Functional MRI Data Using Independent Component Analysis', Human Brain Mapping, 14:140-151.
[5] Biswal, B et al. (1995), 'Functional Connectivity in the Motor Cortex of Resting Human Brain Using Echo-Planar MRI', MRM, 34,:537-541.
[6] Filippini, N. et al. (2009), "Distinct patterns of brain activity in young carriers of the APOE-ε4 allele", PNAS, 106:7209-7214
